# Brensocatib, an oral, reversible inhibitor of dipeptidyl peptidase 1, mitigates interferon-α-accelerated lupus nephritis in mice

**DOI:** 10.3389/fimmu.2023.1185727

**Published:** 2023-06-27

**Authors:** Kuan-Ju Chen, Jimin Zhang, Daniel LaSala, Jessica Basso, Donald Chun, Yuchen Zhou, Patrick P. McDonald, Walter R. Perkins, David C. Cipolla

**Affiliations:** Research Department of Insmed Incorporated, Bridgewater, NJ, United States

**Keywords:** brensocatib, neutrophil serine proteases (NSP), lupus nephritis (LN), interferon-alpha (IFNα), systemic lupus erythematosus (SLE), DPP1 inhibitor, dipeptidyl peptidase 1 (DPPI)

## Abstract

Neutrophils have been implicated in initiating and perpetuating systemic lupus erythematosus and the resultant kidney damage in lupus nephritis (LN) patients, in part through an excessive release of neutrophil serine proteases (NSPs). NSP zymogens are activated by dipeptidyl peptidase 1 (DPP1) during neutrophil maturation and released by mature neutrophils in response to inflammatory stimuli. Thus, a potential strategy to attenuate disease progression in LN would be to inhibit DPP1. We tested whether brensocatib, a highly selective and reversible DPP1 inhibitor, could mitigate LN progression in an interferon-alpha (IFNα)-accelerated NZB/W F1 mouse model. To confirm brensocatib’s pharmacodynamic effect on NSPs in this mouse strain, repeated dose studies were conducted for 7 and 14 days in naïve NZB/W F1 mice *via* oral gavage twice a day. Brensocatib at 2 and 20 mg/kg/day achieved a significant reduction in bone marrow NSP activities after 7 days of daily administration. To initiate LN disease progression, the mice were injected with an IFNα-expressing adenovirus. After 2 weeks, three brensocatib doses (or vehicle) were administered for 6 more weeks. Throughout the 8-week study, brensocatib treatment (20 mg/kg/day) significantly reduced the occurrence of severe proteinuria compared to the vehicle control. Brensocatib treatment also entailed a significant reduction in the urine albumin-to-creatinine ratio, indicating decreased kidney damage, as well as a significant reduction in blood urea nitrogen level, suggesting improved renal function. Based on kidney histopathology analysis, brensocatib treatment significantly lowered both the renal tubular protein score and the nephropathy score compared to the vehicle group. A trend towards reduced glomerulonephritis score with brensocatib treatment was also observed. Lastly, brensocatib significantly reduced LN mouse kidney infiltration by various inflammatory cells. In conclusion, these results suggest that brensocatib alters disease progression in LN mice and warrant further evaluation of DPP1 inhibition in LN.

## Introduction

Systemic lupus erythematosus (SLE) is a systemic autoimmune disease in which the body’s defense system attacks healthy tissues and organs (1, 2). One of the most common and severe manifestations of SLE is lupus nephritis (LN), which is a form of glomerulonephritis. Most patients with SLE develop LN within 5 years of diagnosis (3). Treatments for LN usually involve immunosuppressive therapy including glucocorticoids, cyclophosphamide, mycophenolate mofetil, methotrexate, and azathioprine to reduce morbidity and reduce the risk of end-stage renal disease. However, these treatments are often not uniformly effective (4, 5) and there are significant comorbidities associated with immunosuppressive treatment (6). Within 10 years of initial diagnosis, approximately 5% to 20% of patients with LN develop end-stage kidney disease leading to kidney failure. Despite significant advances in our understanding of LN pathogenesis over the years, LN remains a major challenge (7, 8). Early identification of LN flares and therapies to minimize the incidence and duration of flares is the ultimate goal in LN treatment.

Neutrophils have been implicated in initiating and perpetuating SLE in LN patients, as well as the resultant kidney damage, by triggering profound abnormalities in both innate and adaptive immunity (9–12). One potential treatment strategy is to block the deleterious effects of neutrophil serine proteases (NSPs) released by hyperactivated neutrophils during LN pathogenesis (13). NSPs, which include neutrophil elastase (NE), proteinase 3 (PR3), and cathepsin G (CatG), are synthesized as inactive proproteins during granulocyte development (14). Dipeptidyl peptidase 1 (DPP1, also known as cathepsin C) is the key enzyme that converts pro-NSPs to active NSPs during the neutrophil maturation process in the bone marrow (15, 16). *In vivo* data suggest that pharmacological inhibition of proinflammatory serine proteases may suppress or attenuate deleterious effects of inflammatory and autoimmune disorders mediated by these proteases (17, 18), but evidence specific to LN has not yet been generated. Additionally, NETosis, a unique form of neutrophil cell death that results in the formation of neutrophil extracellular traps (NETs) decorated with NSPs, has also been associated with pathogenesis of SLE and LN (19–21). Thus, a treatment that inhibits NSP activity represents a promising therapeutic approach in tackling LN.

Brensocatib is a small-molecule, competitive, and reversible inhibitor of DPP1 that selectively inhibits NSP activation. It is currently being investigated in a Phase III clinical trial (ASPEN; NCT04594369) in non-cystic fibrosis bronchiectasis (NCFBE). Results from a Phase II study (WILLOW; NCT0321891) in patients with NCFBE demonstrated that brensocatib treatment reduced NE activity in sputum in a dose-dependent manner and prolonged the time to first exacerbation compared to placebo (22). A similar dose-dependent reduction was also observed for PR3 and CatG activity with positive correlations between the three biomarkers in sputum (23, 24). Thus, inhibiting DPP1 may reduce tissue damage resulting from the release of active NSPs by inflammatory neutrophils; likewise, NET reactivity could be attenuated by the reduction in active NSPs present on the NETs.

The involvement of type I interferons (IFNs) in SLE pathogenesis has been suggested in various reviews (25–28). More direct evidence of a role for IFNs in SLE was studied extensively via lupus-prone mice that are either genetically modified to induce type I IFN signaling or stimulated with exogenous type I IFNs (29–31). Treatment with exogenous IFNα accelerates disease progression in lupus-prone mice and has become a useful model for testing potential therapeutics for SLE. Among the various IFNα-accelerated lupus mouse models, the F1 hybrid between the New Zealand black and New Zealand white mouse (NZB/W F1) is widely used. These lupus-prone mice mimic human lupus in several aspects including gender specificity, the appearance of circulating anti-dsDNA antibodies, renal deposition of immune complexes, and the development of fatal glomerulonephritis; however, these mice do not develop skin disease or hematologic manifestations (32, 33). Therefore, this model has primarily been used to investigate therapeutic interventions in LN. In this study, we explored whether brensocatib could mitigate LN progression in these IFNα-accelerated NZB/W F1 mice.

## Materials and methods

### Animal models

A 7-day or 14-day pharmacodynamic (PD) study was conducted in female lupus-prone F1 hybrid between the New Zealand Black (NZB) and New Zealand White (NZW) (NZB/W F1) mice at 10 weeks of age (the Jackson Laboratory, JAX). Brensocatib was formulated in a solution of 0.5% hydroxypropyl methylcellulose (HPMC) in sodium citrate buffer with 0.1% Tween 80 at pH 3.0. Brensocatib at 0.1, 1, or 10 mg/kg/dose or HPMC vehicle control was given via oral gavage at 10 ml/kg twice-a-day (BID) for 7 or 14 days (*n* = 5 per group). At the end of the study, two femurs and two tibias per mouse were collected for bone marrow sample processing and NSP activity measurement. The study was carried out under the guidance of the Institutional Animal Care and Use Committee (IACUC) of the University of Rutgers Animal Care Committee.

Accelerated LN was developed in female lupus-prone NZB/W F1 mice between 10 and 11 weeks of age (JAX) with an injection of IFNα5-expressing adenovirus (IFNα5 Adv) (Vector Biolabs) through the tail vein at 3 × 10^9^ viral particles/mouse; this was followed by 8 weeks of disease progression at which time the animals were sacrificed. Mice were randomized to receive either one of three doses of brensocatib, vehicle control, or anti-mouse interferon alpha/beta/omega receptor subunit 1 antibody (Anti-IFNAR; BioXcell, clone MAR1-5A3) (*n* = 16 per group). Brensocatib at 0.1, 1, or 10 mg/kg/dose or HPMC vehicle control was initiated 2 weeks after virus injection *via* oral gavage BID at 10 ml/kg while Anti-IFNAR was initiated 3 weeks after virus injection via intraperitoneal injection twice per week at 5 ml/kg. Urine collection in metabolic cages and blood collection were performed at baseline and every 2 weeks until the end of the study. All the procedures related to animal handling, care and the treatment in the study were performed according to the guidelines approved by the IACUC of Invivotek, LLC following the guidance of the Association for Assessment and Accreditation of Laboratory Animal Care (AAALAC).

### Proteinuria, urine albumin and creatine, and blood urea nitrogen measurement

Proteinuria values were separately assessed in urine every week by ChemStick to monitor proteinuria progression. Albumin was measured in mouse urine samples using the Mouse Albumin ELISA Quantitation Set from Bethyl Laboratories (Montgomery, Texas). Following the manufacturer’s instructions, each sample was run at more than one dilution, each with and without a spike of mouse albumin standard to test for the percent recovery. HRP detection antibody was used at a dilution of 1:20,000. Creatinine was measured for each sample using the Exocell Creatinine Companion colorimetric assay kit from Ethos Biosciences (Logan Township, NJ) following the manufacturer’s instructions with the exception that the calibrator was run as a twofold serial dilution starting at 10 mg/dl. Urine samples were first diluted 20-fold and 20 µl was used for the assay. Albumin concentrations were divided by creatinine to yield the albumin/creatinine ratio of µg albumin per mg creatinine. Blood urea nitrogen (BUN) was assessed using the QuantiChrom Urea Assay Kit (BioAssay).

### Bioanalytical analysis

For plasma collection for pharmacokinetic (PK) analysis, blood was collected via the tail-vein technique 2 h post-dose, except for week 8, where the plasma was collected 16 h post-final dose before euthanasia. The samples were centrifuged and stored at −80°C until drug measurement by LC-MS/MS as previously described [ (34); Basso et al. The PK profile of brensocatib and its effect on PD biomarkers (NE, PR3, and CatG) in various rodent species. Submitted for publication].

### Histology

Left kidneys were used for preparing Periodic acid–Schiff (PAS) and H&E-stained slides, and histopathology was assessed by a certified veterinary pathologist for individual scores of three characteristics: tubular protein, nephropathy, and glomerulonephritis, as well as their sum scores. The grading or scoring is defined as follows: 0, none or normal; 1, minimal; 2, mild; 3, moderate; 4, severe. Representative images for each scoring are shown in **Figure S3**. Treatment information of the mice was blinded to the pathologist.

### Infiltrated inflammatory cell quantification

Right kidneys were used for tissue digestion and Percoll centrifugation, followed by flow cytometry with fixable viability dye staining and surface staining for CD45, CD11b, Ly6G, Ly6C, F4/80, and CD3 as previously described (30). Analysis of each subtype was based on the analyses described in various publications (35, 36) where leukocytes were defined by the CD45^+^ population, myelocytes were defined by the CD45^+^CD11b^+^ population, and T lymphocytes were defined by the CD45^+^CD3^+^ population. The CD45^+^CD11b^+^ population was further characterized using the markers Ly6G for neutrophils, Ly6C for the monocyte subset, and Ly6C and F4/80 for the macrophage subset. Flow cytometry data were analyzed with FlowJo version 10.8.1.

### Bone marrow collection

Bone marrow cells were collected from two tibias and two femurs from each animal of the naïve NZB/W F1 mice. In brief, bone cutters were used to cut the epiphysis (knobby ends) off the long bones to expose the bone marrow-filled cavity. Each bone cavity was then flushed with 5 ml/bone of ice-cold RPMI media through a 40 μM cell strainer and collected in a 50-ml conical tube. Cells were spun down at 2,500 ×*g* at 4°C for 5 min and the supernatant was aspirated. After which, bone marrow cells were lysed with red blood cell (RBC) lysis buffer to eliminate contaminating RBCs and washed with PBS to remove any residual RBC lysis buffer. The resulting white blood cells (WBCs) were subsequently lysed with 1% (v/v) Triton X-100 in PBS. Lysates were added to 96-well plates and stored at −80°C until NSP analyses.

### NSP activity measurement

Bone marrow WBC lysate collected from the naïve NZB/W F1 mice was assayed for NSP activities using enzymatic kinetic assays described previously (37). Peptide substrates for NE (N-Methoxysuccinyl-Ala-Ala-Pro-Val-7-amido-4-methylcoumarin; Sigma; St. Louis, MO; Excitation/Emission at 350/450 nm), PR3 [(7-Methoxycoumarin-4-yl)acetyl-lysyl-(picolinoyl)-Tyr-Asp-Ala-Lys-Gly-Asp-N-3-(2-4-dinitrophenyl)-2-3-diaminopropyonyl-NH2; GenScript; Piscataway, NJ; excitation/emission at 340/430 nm], and CatG (N-Succinyl-Ala-Ala-Pro-Phe p-nitroanilide; Sigma; Absorbance at 405 nm) were added at a final assay concentration of 100 μM, 40 μM, and 200 μM, respectively, and either fluorescence or absorbance was quantified using a Synergy microplate reader (BioTek; Winooski, VT). The specific NSP activity in each sample was calculated as the total activity minus the activity in the presence of a specific NSP inhibitor (if used), including elastase inhibitor (Abcam) for NE, sivelestat (Abcam) for PR3, and cathepsin G inhibitor I (Cayman Chemical; Ann Arbor, MI) for CatG. Active NSP concentrations were interpolated based on their activities relative to the standard curves created using active human NE protein (Sigma), active human PR3 protein (Sigma), and active human CatG protein (Sigma), respectively. Owing to the unavailability of commercial mouse NE, PR3, and CatG proteins, the corresponding human proteins were used instead since their catalytic ability is considered to be conserved across species. A portion of each cell lysate sample was retained for protein quantification using a Pierce™ BCA Protein Assay Kit (Thermo Fisher). The NSP activities were normalized for the cell lysate protein concentrations.

### Statistical analyses

Statistical analyses were performed by one-way ANOVA followed by a Dunnett *post-hoc* test, or Log-rank (Mantel-Cox) test (for analysis of progression curve to proteinuria > 750 mg/dl), with a significance threshold of *p* < 0.05. Values presented are mean ± SEM. NSP activity was analyzed by two-way ANOVA followed by a Sidak’s *post-hoc* test.

## Results

### Pharmacodynamic effect of brensocatib in lupus-prone NZB/W F1 mice

**Figure 1:**
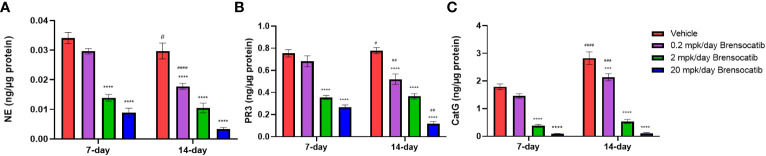
Pharmacodynamic effect of brensocatib in NZB/W F1 mice following BID dosing over 7 and 14 days. Brensocatib was administered at 0.2, 2, and 20 mg/kg/day via oral gavage. Bone marrow samples were collected approximately 16 h after the final dose. NSP activities were measured in the bone marrow samples: **(A)** NE, **(B)** PR3, and **(C)** CatG. Data are plotted as mean ± SEM (*n* = 5/group). * indicates data that are significantly different (***, *p* < 0.001; ****, *p* < 0.0001) from the vehicle control within the same dose duration; # indicates data that are significantly different (##, *p* < 0.01; ###, *p* < 0.001; ####, *p* < 0.0001) between dose durations for respective doses. mpk/day = mg/kg/day.

To investigate the effect of brensocatib on NSP activities in lupus-prone NZB/W F1 mice, we evaluated repeated brensocatib BID dosing for either 7 or 14 days. The resulting bone marrow NSP activities are shown in **Figure 1**. When compared to the appropriate vehicle control, a dose-dependent reduction in bone marrow NSP levels was observed for all three NSPs. At 20 mg/kg/day brensocatib, NE, PR3, and CatG were reduced by 74% (*p* < 0.0001), 64% (*p* < 0.0001), and 95% (*p* < 0.0001) after 7-day dosing; and by 89% (*p* < 0.0001), 85% (*p* < 0.0001), and 96% (*p* < 0.0001) after 14-day dosing, respectively. The results confirmed that a significant reduction in NSP activities can be achieved after 7 days of treatment; more than 85% of NSP activities were inhibited after 14 days of dosing.

### Pharmacokinetic profile of brensocatib in Adv-IFNα5-treated NZB/W F1 mice

**Figure 2:**
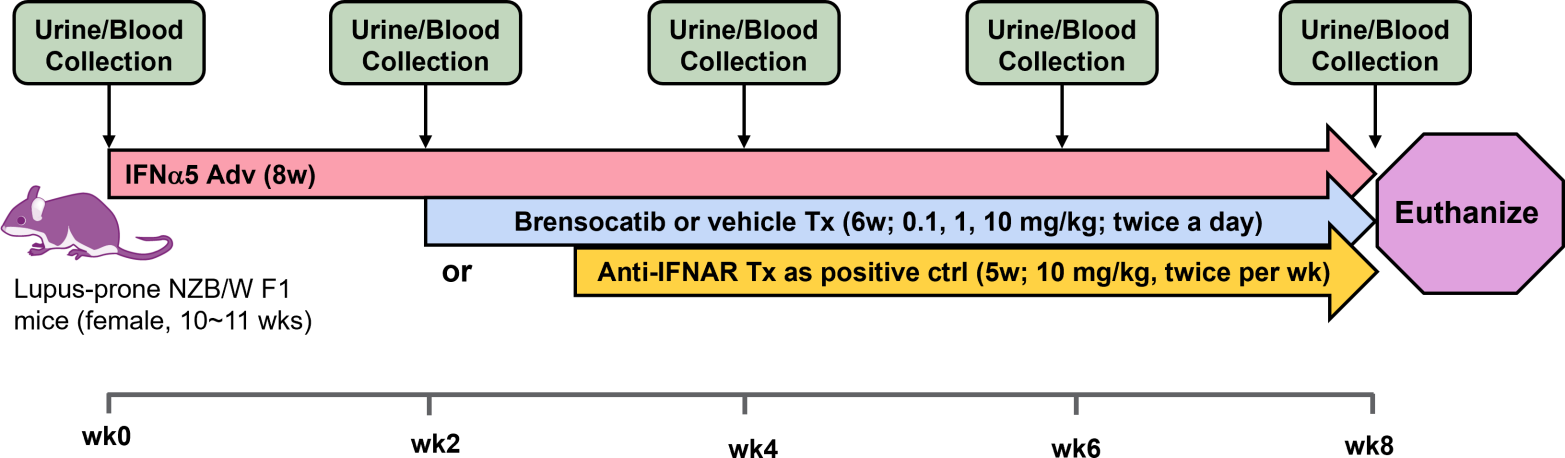
Schematic representation of the study design for the IFNα-accelerated LN mouse model. Female lupus-prone NZB/W F1 mice were injected with IFNα5-expressing adenovirus (IFNα5 Adv) through the tail vein followed by 8 weeks of disease progression. Two weeks after virus injection, brensocatib (0.1, 1, and 10 mg/kg) or vehicle was administered BID via oral gavage while anti-mouse interferon alpha/beta/omega receptor subunit 1 antibody (Anti-IFNAR) was initiated 3 weeks after virus injection twice per week via intraperitoneal injection. Urine collection in metabolic cages and blood collection were performed at baseline and every 2 weeks to monitor disease progression.

**Figure 3:**
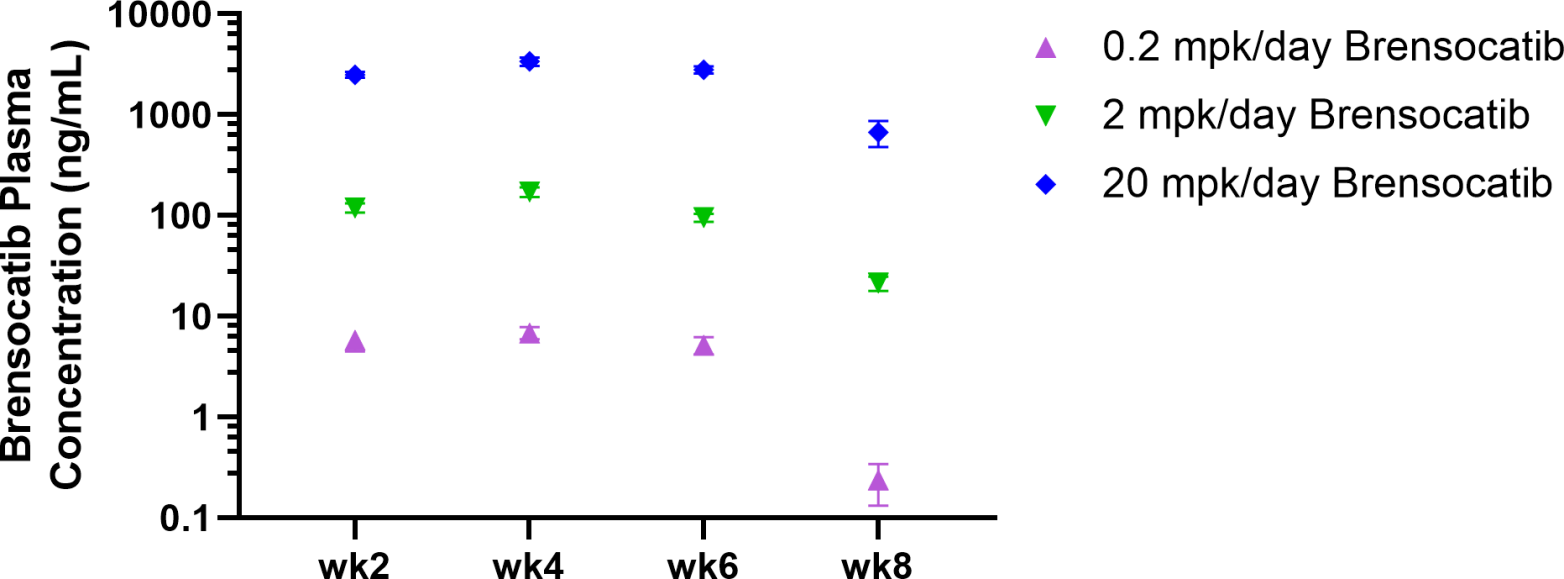
Brensocatib plasma concentration throughout the treatment course in the IFNα-accelerated LN mouse model. Plasma was collected 2 h post-dose (Cmax) except for week 8, where plasma was collected 16 h post-final dose before euthanasia. Data are plotted as mean ± SEM (*n* = 16 per group except *n* = 15 for 0.2 mpk/day brensocatib at week 6 and 2 mpk/day brensocatib at week 8; *n* = 13 for 0.2 mpk/day brensocatib at week 8). mpk/day = mg/kg/day.

The study timeline to investigate brensocatib in the accelerated LN model was 8 weeks (**Figure 2**). Brensocatib was administered to the mice 2 weeks after the injection of IFNα5 Adv. To monitor drug exposure in these mice, the brensocatib concentration in plasma was monitored every 2 weeks throughout the course of the study. Plasma samples were collected 2 h after the brensocatib afternoon dose for weeks 2, 4, and 6, which represents the timing for Cmax, and 16 h post-last dose for week 8. Consistent and dose-dependent exposure for the three dose groups was observed; the results are summarized in **Figure 3**. The average plasma drug concentration from the week 2 to week 6 measurements was 5.96 ng/ml, 129.3 ng/ml, and 2,809 ng/ml for brensocatib treatment at 0.2 mg/kg/day, 2 mg/kg/day, and 20 mg/kg/day, respectively. Importantly, no adverse side effects of brensocatib treatment were observed for these doses in terms of animal body weight loss, abnormal behavior signs, and appearance changes (data not shown). Overall, brensocatib demonstrated a dose-dependent effect and convincing safety profile.

### Disease progression and animal pseudo survival rate

**Figure 4:**
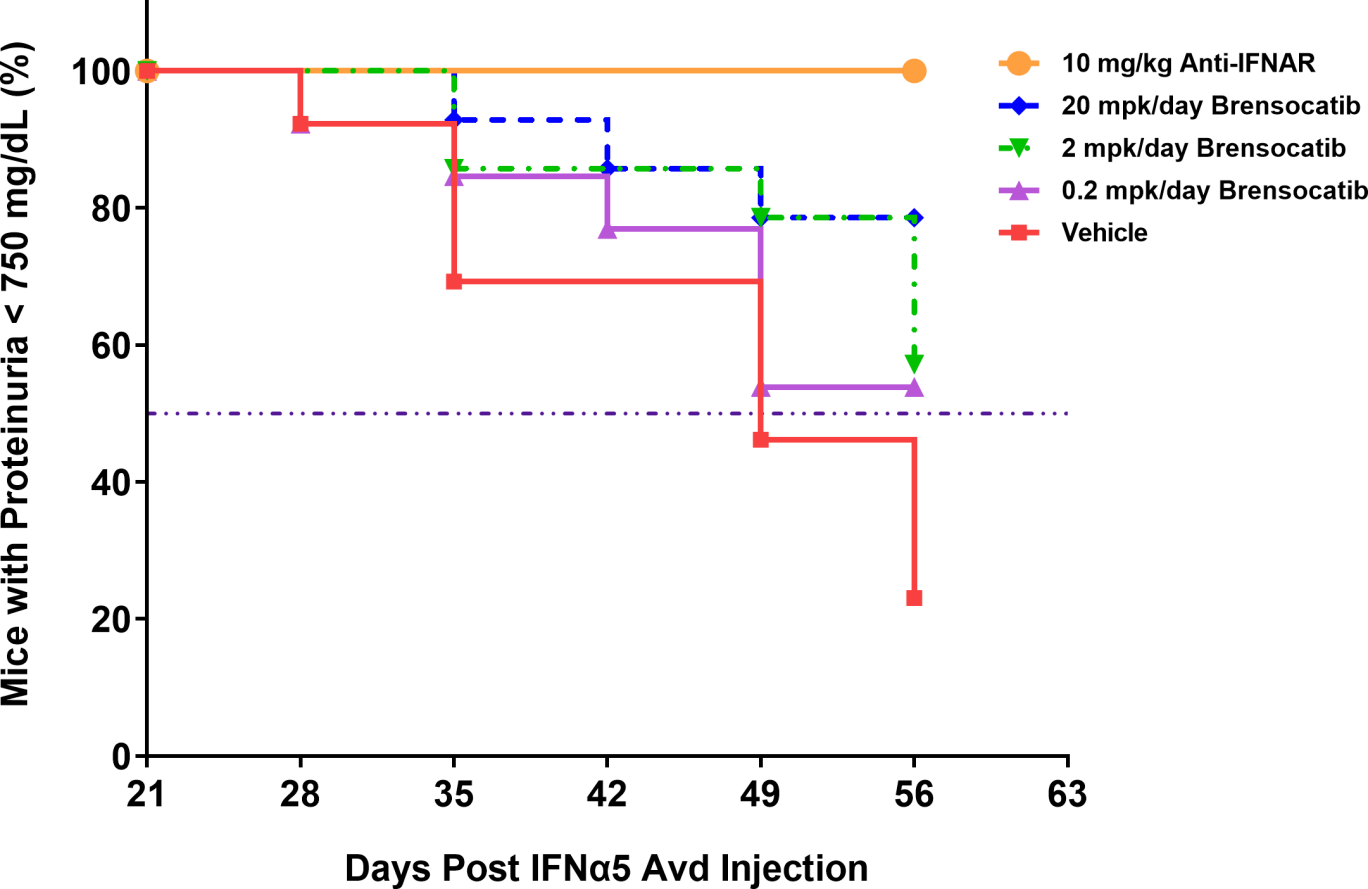
Disease progression in the IFNα-accelerated LN mouse model was quantified by the percentage of animals with proteinuria less than 750 mg/dl (*n* = 16 per group). The majority of NZB/W F1 mice with IFNα5 Adv injection progressed into severe proteinuria (>750 mg/dl) starting on day 28 in the vehicle group while brensocatib treatment at 20 mg/kg/day significantly delayed the progression to day 56 compared to the vehicle control (Log-rank analysis, *p* = 0.0107). Anti-IFNAR treatment as the positive control group exhibited no severe proteinuria until day 56 (Log-rank analysis, *p* < 0.0001), which was the expected outcome from abolishing IFNα signaling. Broken solid line is set at 50%. mpk/day = mg/kg/day.

**Figure 5:**
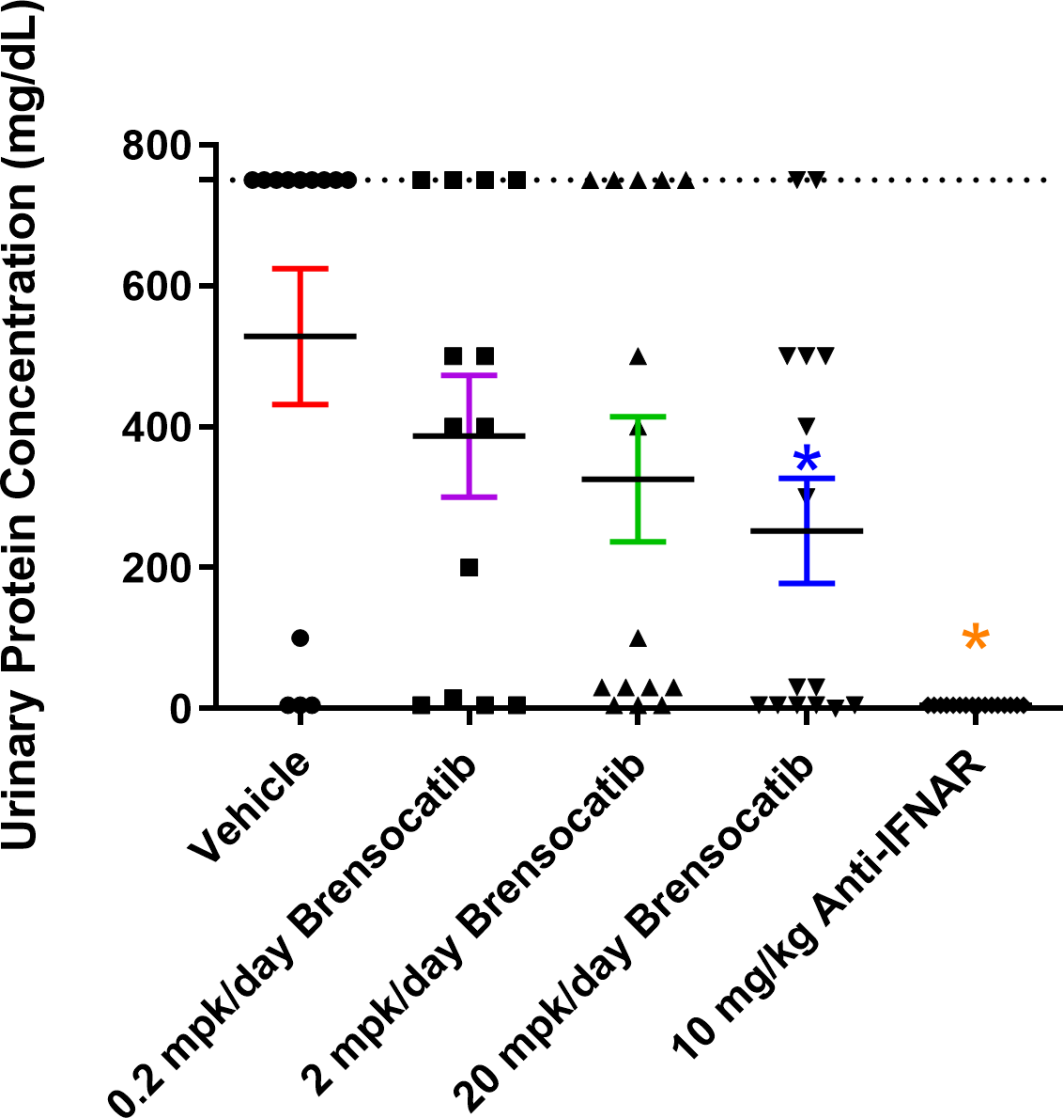
Measurement of urine protein via ChemStick from IFNα-accelerated LN mice at week 8. Mice were placed in metabolic cages for 24-h urine collection and analyzed for protein content. Data are plotted as mean ± SEM (*n* = 16 for 10 mg/kg Anti-IFNAR; *n* = 13 for the vehicle and 0.2 mpk/day brensocatib; *n* = 15 for 2 mpk/day brensocatib and 20 mpk/day brensocatib). *, *p* < 0.05 *vs*. vehicle. Dotted line is set at 750 mg/dl. mpk/day = mg/kg/day.

The most common manifestation of LN is proteinuria; as a result, its measurement was used to monitor disease progression as well as to determine a pseudo survival rate throughout the study. The Kaplan–Meier plot in **Figure 4** summarizes the percentage of mice that had severe proteinuria occurrence (defined as > 750 mg/dl) in each group. On day 28 post-IFNα5 Adv injection, mice from the vehicle group began to progress into severe proteinuria. The median time of occurrence for the vehicle group was 49 days while none of the brensocatib treatment arms reached the median time to severe proteinuria through the end of the study at day 56 (Log-rank analysis, *p* = 0.0107 for 20 mg/kg/day compared to vehicle). Rodents treated with the positive control (Anti-IFNAR), expected to abolish IFNα signaling, exhibited no severe proteinuria (Log-rank analysis, *p* < 0.0001) through the end of the study. The amount of protein in the urine on week 8 increased to 528 ± 86.8 mg/dl (mean ± SEM) for the vehicle control. In contrast, the brensocatib groups all had lower urine protein; in particular, there was a statistically significant decrease to 252 ± 72 mg/dl in the 20 mg/kg/day brensocatib treatment group, relative to the vehicle control (**Figure 5**). Proteinuria is a major risk factor of renal disease progression, and it plays a critical role in damaging renal function. The delayed disease progression observed with brensocatib treatment when measuring proteinuria indicates that DPP1 inhibition has therapeutic potential.

### Renal damage and function assessment in urine and blood samples

**Figure 6:**
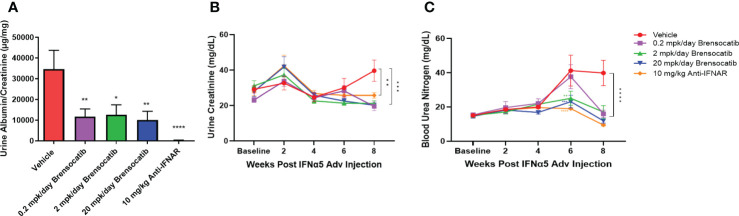
Kidney damage and renal function assessment. **(A)** Kidney damage is assessed by the ratio of albumin to creatinine in urine at week 8. Mice treated with brensocatib exhibited a statistically significant reduction in this marker of kidney damage. **(B)** Time course measurement of urine creatinine via Exocell creatinine companion colorimetric assay kit at weeks 0, 2, 4, 6, and 8 weeks. **(C)** Renal function is monitored by blood urea nitrogen in blood at weeks 0, 2, 4, 6, and 8. Brensocatib at the two higher doses significantly reduced blood urea nitrogen compared to the vehicle group at week 6 while all three brensocatib doses showed improved renal function at week 8. Data are plotted as mean ± SEM (*n* = 16 per group except *n* = 15 for vehicle group at weeks 4, 6, 0.2 mpk/day brensocatib at week 6, and 2 mpk/day brensocatib and 20 mpk/day brensocatib week 8; *n* = 13 for vehicle group, 0.2 mpk/day brensocatib at week 8). *, *p* < 0.05; **, *p* < 0.01; ***, *p* < 0.001; ****, *p* < 0.0001 *vs*. vehicle. mpk/day = mg/kg/day.

To investigate the ability of brensocatib to impede the progression of renal damage, albumin was measured on week 8 and creatinine was measured on weeks 0, 2, 4, 6, and 8 in urine samples. No differences were observed in urine creatinine concentration for up to 6 weeks post IFNα5 Adv injection among all groups. The vehicle group had increased creatinine concentration in urine at week 8 and brensocatib treatment at all three doses reduced the urine creatinine level significantly compared to the vehicle (*p* = 0.0001 for 0.2 mg/kg/day and 20 mg/kg/day; *p* = 0.0003 for 2 mg/kg/day; **Figure 6B**). The urine albumin-to-creatinine ratio of each group measured on week 8 is shown in **Figure 6A**. Brensocatib at all three doses had similar albumin-to-creatinine ratios and were significantly lower than the vehicle control. Plasma BUN is a common indicator of kidney function. Plasma samples were collected on weeks 0, 2, 4, 6, and 8 to monitor the disease progression (**Figure 6C**). At week 6, the BUN level was elevated in the vehicle group but brensocatib treatment (at 2 mg/kg/day and 20 mg/kg/day) significantly blunted the rise in BUN level, suggesting that brensocatib treatment improved kidney function in this IFNα-accelerated lupus mouse model. At week 8, all three brensocatib treatment doses exhibited statistically significant improvements in renal function compared to the vehicle. The positive control, the Anti-IFNAR treatment group, also exhibited a low albumin-to-creatinine ratio and BUN level compared to the vehicle group. Thus, brensocatib at all three doses reduced kidney damage and improved renal function at the end of the study.

### Histology analysis of kidney tissues

**Figure 7:**
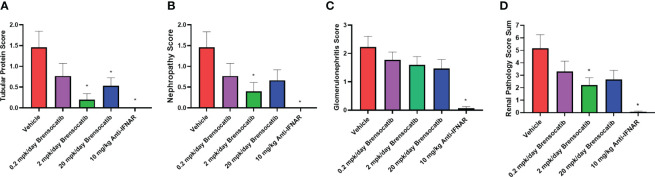
Kidney histopathological scores related to **(A)** tubular protein, **(B)** nephropathy, **(C)** glomerulonephritis, and **(D)** overall renal pathology. Brensocatib treatment at 2 mg/kg/day significantly decreased the renal histopathological scores of tubular protein, nephropathy, and overall renal pathology score. A trend towards reduced glomerulonephritis score was also observed with brensocatib treatment. Data are plotted as mean ± SEM (*n* = 13 for vehicle and 0.2 mpk/day brensocatib; *n* = 15 for 2 and 20 mpk/day brensocatib; *n* = 16 for Anti-IFNAR). *, *p* < 0.05 *vs*. vehicle. mpk/day = mg/kg/day.

Blinded analysis of renal histology by an independent pathologist demonstrated an increase in tubular protein (A), nephropathy (B), and glomerulonephritis (C) in the IFNα-accelerated NZB/W F1 mice (**Figure 7**). The two higher doses of brensocatib (2 and 20 mg/kg/day) decreased the tubular protein score compared to the vehicle control; however, no dose dependency was observed (**Figure 7A**). Brensocatib treatment also led to a lower nephropathy score compared to the vehicle group but only the 2 mg/kg/day group was considered statistically significant (**Figure 7B**). Even though a trend towards decreased glomerulonephritis score was observed, no statistically significant improvement was noted for any of the brensocatib doses (**Figure 7C**). Together, a sum of the three renal pathology scores is shown in **Figure 7D**. Both brensocatib at 2 mg/kg/day and the Anti-IFNAR treatment demonstrated a significant decrease in overall kidney pathology score compared to the vehicle.

### Inflammatory cell quantification in kidney tissues

**Figure 8:**
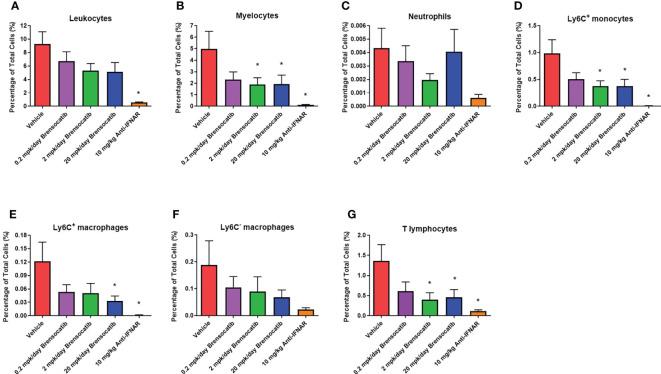
Percentage of various inflammatory cells in the kidney measured by flow cytometry, including **(A)** leukocytes, **(B)** myelocytes, **(C)** neutrophils, **(D)** Ly6C^+^ monocytes, **(E)** Ly6C^+^ macrophages, **(F)** Ly6C^-^ macrophages, and **(G)** T lymphocytes. Brensocatib treatment significantly reduced the amount of myelocytes, Ly6C^+^ monocytes, Ly6C^+^ macrophages, and T lymphocytes in the kidney but not the population of neutrophils compared to the vehicle control. Data are plotted as mean ± SEM (*n* = 13 for vehicle and 0.2 mpk/day brensocatib; *n* = 15 for 2 and 20 mpk/day brensocatib; *n* = 16 for Anti-IFNAR). *, *p* < 0.05 *vs*. vehicle. mpk/day = mg/kg/day.

Inflammatory cell infiltration into the kidney was assessed via flow cytometry (representative flow cytometry plots for each group are shown in **Figure S1, S2**). These analyses showed a significant increase in the percentage of various immune cells within the kidney after the injection of IFNα5 Adv (**Figure 8**). Based on the gating strategy, brensocatib did not have a significant impact on total infiltrating leukocytes (CD45^+^ cells; **Figure 8A**), but brensocatib at the two higher doses reduced infiltration of myelocytes (CD45^+^CD11b^+^ cells; **Figure 8B**) and T lymphocytes (CD45^+^CD3^+^ cells; **Figure 8G**) compared to the vehicle-treated group. Further analyses of the CD45^+^CD11b^+^ population suggested that brensocatib significantly reduced CD45^+^CD11b^+^Ly6C^+^ monocyte infiltration in the kidney at both the 2 mg/kg/day and 20 mg/kg/day doses (**Figure 8D**) but had no effect on the neutrophil population (CD45^+^CD11b^+^Ly6G^+^ cells; **Figure 8C**). The Anti-IFNAR treatment group demonstrated significant reductions in inflammatory cell infiltration percentages for leukocytes, myelocytes, CD3^+^ T lymphocytes, and Ly6C^+^ monocytes. In the case of macrophages (**Figures 8E, F**), both brensocatib at the highest dose and the Anti-IFNAR treatment group significantly reduced Ly6C^+^ macrophages and showed a decreased trend in the Ly6C^-^ macrophage population compared to the vehicle control. In conclusion, brensocatib treatment significantly reduced the amount of myelocytes, Ly6C^+^ monocytes, Ly6C^+^ macrophages, and T lymphocytes in the kidney while the population of neutrophils remained unaffected compared to vehicle control.

## Discussion

Brensocatib’s PK profile and PD effect on the three NSPs (i.e., NE, PR3, and CatG) have been studied extensively in naïve rodents including C57BL/6 mice, BALB/c mice, SD rats, and Wistar rats [ (34); Basso et al. The PK profile of brensocatib and its effect on PD biomarkers (NE, PR3, and CatG) in various rodent species. Submitted for publication]. Dose-dependent PK exposure responses (AUC and Cmax) with a short half-life of 2–4 h were observed in those rodent species and strains. In the same animals, a maximum reduction in NSP activities was observed after ∼7 days of brensocatib treatment. However, there were variations among species and strains regarding both the extent and the time to reach the maximum reduction for each NSP. To understand brensocatib’s PD effect in NZB/W F1 female mice, dosing of brensocatib BID for 7 and 14 days was conducted. A dose-dependent response was observed for both treatment durations. Brensocatib treatment for 7 days at both 2 and 20 mg/kg/day achieved a significant reduction in all three NSP activities isolated from the bone marrow. A slightly greater reduction in NE and PR3 activities was observed after the 14-day treatment duration. Interestingly, brensocatib elicited the strongest inhibitory effect against CatG in NZB/W F1 female mice followed by NE and then PR3. This confirms that brensocatib at both 2 mg/kg/day and 20 mg/kg/day achieves a strong reduction in NSP activity in the accelerated LN study.

The NZB/W F1 mouse model is one of the classic models of spontaneous lupus (38). The IFNα-induced model is a refinement of the spontaneous NZB/W F1 model, which accelerates disease progression from months to just weeks, and allows synchronization of disease state across animals (32). In light of the involvement of neutrophils and NSPs in SLE pathogenesis (especially the association with and contribution to kidney damage), we investigated the potential of brensocatib as a therapeutic intervention for LN in the IFNα-accelerated NZB/W F1 model, which was an 8-week study featuring 6 weeks of treatment. Historical data suggest that IFNα5 Adv-treated NZB/W F1 mice start to present lupus-associated manifestations within 3 to 4 weeks and the treatment intervention normally starts on week 3 (39). However, given that it takes a week for brensocatib to reach its full PD effect [ (34); Basso et al. The PK profile of brensocatib and its effect on PD biomarkers (NE, PR3, and CatG) in various rodent species. Submitted for publication], we designed the treatment regimen of brensocatib to start 2 weeks after the injection of IFNα5 Adv and a week before the positive control treatment, i.e., Anti-IFNAR. By monitoring plasmatic brensocatib concentration, the dose-dependent exposure to the drug confirmed that treatment administration was consistent with the study design throughout the course of the study. In this regard, it was expected that brensocatib treatment at 0.2 mg/kg/day may not be inadequate to ameliorate disease progression, given that there was minimal inhibition of NSP activity. Indeed, even though all three brensocatib treatment doses reduced the occurrence of mice that experienced severe proteinuria, only the 20 mg/kg/day brensocatib treatment group demonstrated a statistically significant improvement compared to the vehicle group. As expected, mice exposed to the 5-week Anti-IFNAR treatment at 10 mg/kg/day exhibited minimal proteinuria (5 mg/dl) at the end of the 8-week study.

As an early sign of kidney damage (40), the ratio of albumin to creatinine in urine was measured at week 8. Brensocatib treatment resulted in a less severe disease state in these mice. When monitoring the renal function throughout the study, brensocatib treatment at 20 mg/kg/day decreased the BUN level to a similar extent to that of Anti-IFNAR treatment, and showed significant enhancement compared to the vehicle group. Additionally, brensocatib treatment at 2 mg/kg/day significantly repressed the severity of the kidney histological changes in tubular protein score and the nephropathy score compared to the vehicle, while the glomerulonephritis score was not considerably affected. This may be explained by a small number of increased deaths in the vehicle group—those animals that did not survive to study end were not evaluated for histopathology scoring, potentially introducing bias that favored the vehicle group. Combining the three parameters, brensocatib treatment improved the overall renal pathology score compared to the control.

Immune cell infiltration into the kidney is also a hallmark of LN. Infiltrating cells include myelocytes, monocytes, B cells, and T cells, which together cause imbalances in the cytokine environment. In this regard, both Th1 cytokines (such as IL-2, IL-12, and IFNγ) and Th2 cytokines (IL-4, IL-5, IL-10, and IL-13) have been implicated in SLE pathogenesis and inflammation-related tissue damage in LN. Thus, the ability to inhibit inflammatory cell infiltration as well as to tamp down the resulting cytokines could have a major impact on disease development. A reduction in the infiltration of CD45^+^CD11b^+^ myelocytes, Ly6C^+^ monocytes, and CD3^+^ T lymphocytes was indeed observed in the diseased kidney following brensocatib treatment. It has been demonstrated that DPP1 knockout mice have normal *in vitro* neutrophil chemotaxis and *in vivo* neutrophil accumulation during sterile peritonitis. Additionally, polymorphonuclear neutrophils of the DPP1-deficient mice do not have an intrinsic defect in their ability to transmigrate across the endothelial barrier into the air pouch environment in response to CXC-like chemokines (41). Even though brensocatib only caused a minimal inhibitory effect on the infiltration of mature neutrophils, their remarkably reduced NSP activity may also contribute to attenuate disease progression and tissue damage. Together, our findings provide evidence supporting brensocatib as a new therapeutic option for the treatment of LN.

While brensocatib inhibited the migration of inflammatory cells into the kidney and attenuated the course of renal disease in this IFNα-induced mouse model of LN, there are several important limitations to note. SLE is a multifactorial disease, and its etiology likely involves both genetic and environmental contributions not exclusively related to type I IFN induced loss of tolerance and tissue damage (2). Thus, the improvements observed in the IFNα-induced mouse model of LN with brensocatib treatment may not translate to the human clinical setting. The IFNα-induced mouse model of LN is a relatively recent model (27), and consequently, there are few literature references for therapeutics that have been evaluated in this model (42, 43). However, our positive control, a mouse antibody to the interferon alpha receptor, demonstrated strong efficacy in this model. Additionally, anifrolumab, a human monoclonal antibody that binds to the type I interferon receptor (IFNAR1), met the primary endpoint in one Phase III trial (44, 45) and was recently approved in the US for the treatment of adult patients with moderate to severe SLE who are receiving standard therapy (46). Thus, while there is limited experience with other therapeutic interventions to inform on the clinical translatability of this model, the clinical experience with anifrolumab is supportive. Based on these observations, evaluation of brensocatib in other SLE animal models and an exploratory approach with combination therapy appears warranted.

## Conclusion

The IFNα-accelerated NZB/W F1 mouse LN model was utilized to investigate the potential benefits of DPP1 inhibition on disease progression. By 28 days post-IFNα5 Adv injection, mice started to develop manifestations associated with LN. Vehicle-treated mice exhibited progressive proteinuria, high urine albumin-to-creatinine ratio, elevated BUN levels, and evidence of severe nephritis based on pathology. Treatment of mice with brensocatib, a DPP1 inhibitor, delayed the disease progression to severe proteinuria as compared with those treated with vehicle only. Additional benefits of brensocatib treatment included an improvement in renal function, a decrease in inflammatory cell infiltration into the kidney, and a repression of kidney damage. Overall, treatment with brensocatib demonstrated therapeutic potential in the accelerated LN model, warranting further evaluation of DPP1 inhibition in this indication.

## Data availability statement

The original contributions presented in the study are included in the article/**Supplementary Material**. Further inquiries can be directed to the corresponding author.

## Ethics statement

The animal study was reviewed and approved by The institutional Animal Care and Use Committee (IACUC) of the University of Rutgers Animal Care Committee as well as the IACUC of Invivotek, LLC following the guidance of the Association for Assessment and Accreditation of Laboratory Animal Care (AAALAC).

## Author contributions

K-JC, JZ, WP, and DC contributed to conceptualization. JZ, DL, JB, DC, and YZ performed the experiments. K-JC and JZ analyzed the data. K-JC drafted the manuscript. K-JC, JZ, PM, WP, and DC reviewed and edited the manuscript. All authors contributed to the article and approved the submitted version.
