# Supplementary material for: Brensocatib, an oral, reversible inhibitor of dipeptidyl peptidase 1, mitigates interferon-α-accelerated lupus nephritis in mice
Source: Front Immunol. 2023 Jun 27;14:1185727. doi: 10.3389/fimmu.2023.1185727 (PMC10333524; doi:10.3389/fimmu.2023.1185727)
Supplement: Supplementary file 1 [file DataSheet_1.pdf]

Supplementary Material

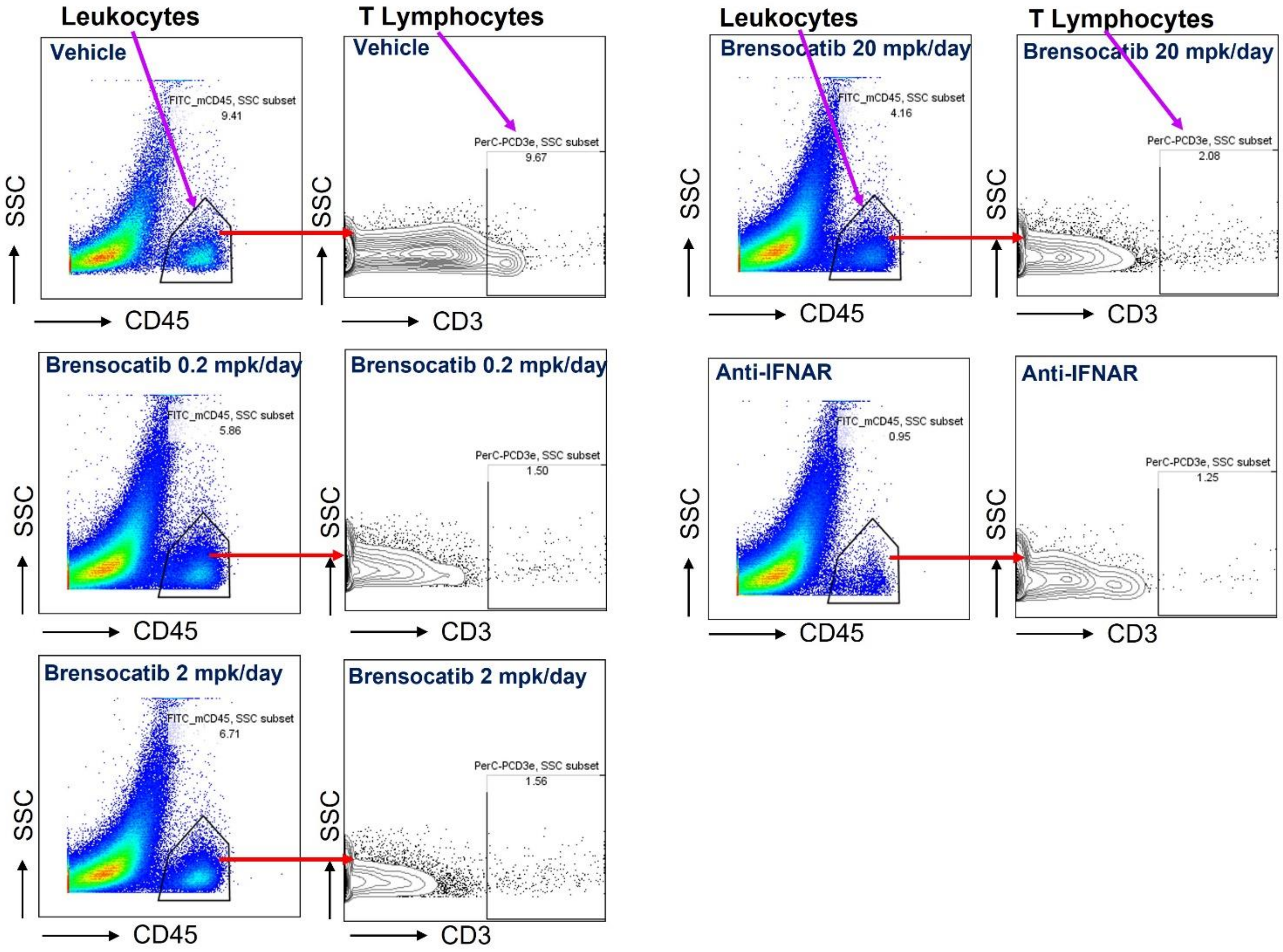

Figure S1. Stepwise gating of CD45+ cells (leukocytes) by flow cytometry as CD45+CD3+ cells (T lymphocytes) from isolated kidney cells from NZB/W F1 mice treated with either vehicle, brensocatib, or anti-IFNAR. Representative flow cytometry plots for each group are shown.

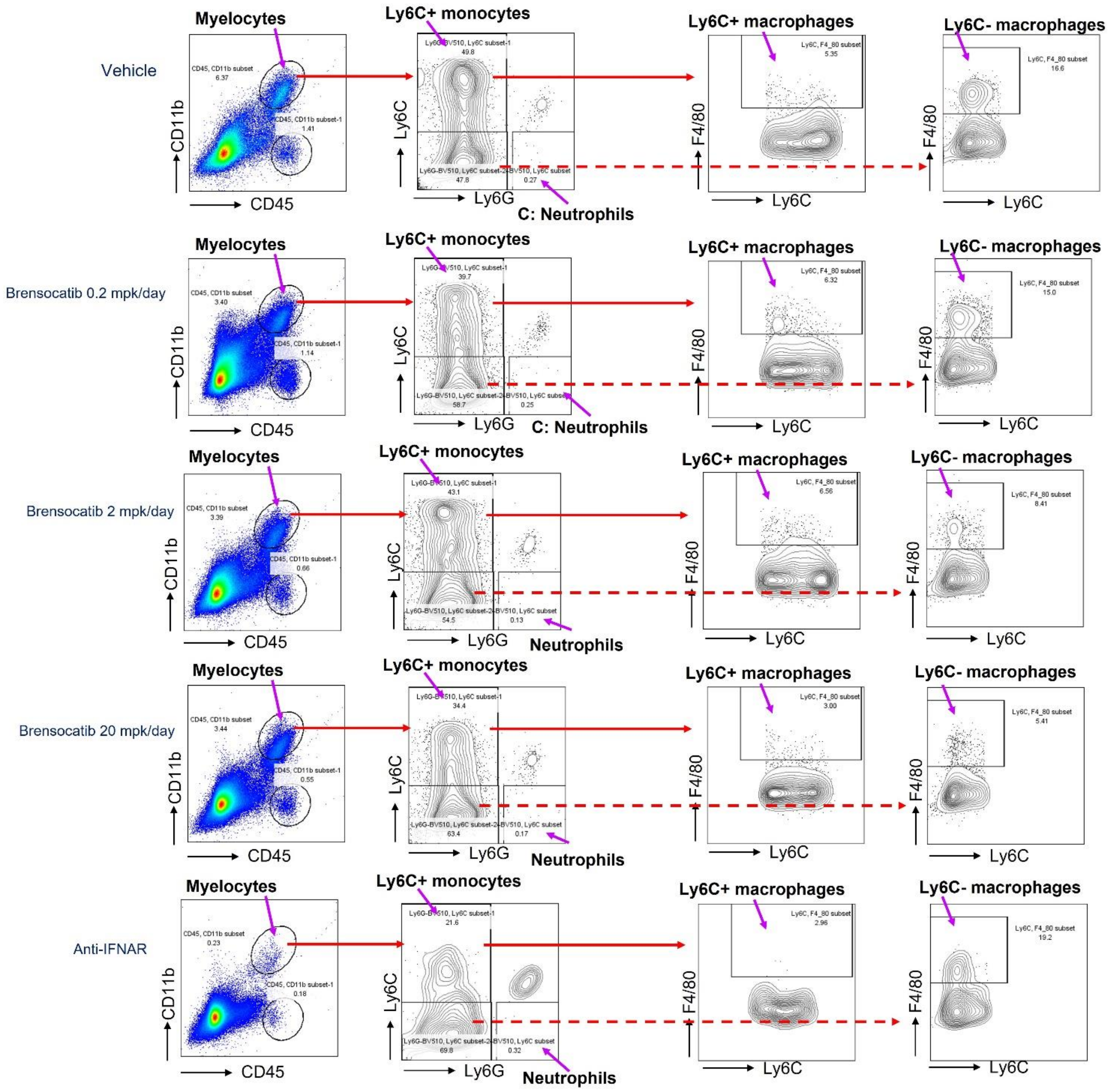

Figure S2. The percentages of renal-infiltrating neutrophils, Ly6C+ monocytes, Ly6C+ macrophages, and Ly6C- macrophages in total renal-infiltrating CD45+CD11b+ cells (myelocytes) of NZB/W F1 mice as determined by flow cytometry. The gating strategy and representative flow cytometry plots for each group are shown.

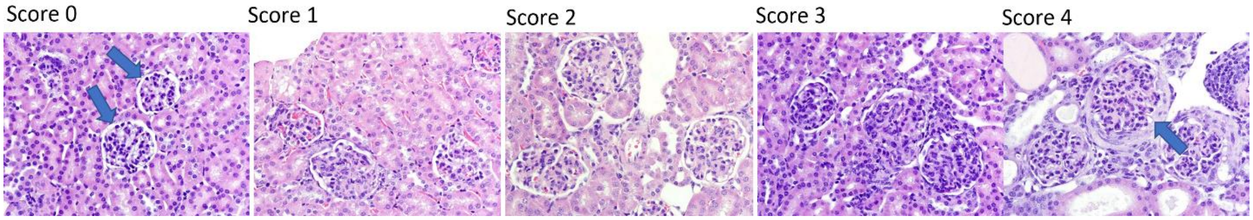

Figure S3. Representative kidney histological images with hematoxylin and eosin staining. Glomerulonephritis scoring is defined: 0, normal glomeruli (arrows); 1, minimal increase in glomerular cellularity and glomerular size; 2, mild increase in glomerular cellularity; 3, moderate increase in glomerular cellularity; 4, Severe degree of glomerular lesions including cellularity, increased mesangial matrix and periglomerular fibrosis (arrow). Note tubular atrophy (upper left) and focus of lymphocytes (upper right). Overall kidney score also influenced by involvement of tubules and presence of tubular casts. 40X magnification.
